# The feasibility and safety of a through-and-through wire technique for central venous occlusion in dialysis patients

**DOI:** 10.1186/s12872-016-0411-3

**Published:** 2016-12-07

**Authors:** Yonghui Huang, Bing Chen, Guosheng Tan, Gang Cheng, Yi Zhang, Jiaping Li, Jianyong Yang

**Affiliations:** The Department of Interventional Radiology, The First Affiliated Hospital, Sun Yat-sen University, 58 Zhongshan Road II, Guangzhou, 510080 China

**Keywords:** Central venous occlusion disease, Hemodialysis, Endovascular intervention, Stent, Though-and-through

## Abstract

**Background:**

To retrospectively compare the operation time, success rate and efficacy between unidirectional and bidirectional procedures in the treatment of central venous occlusion diseases (CVOD), assess the advantages of the bidirectional approach, and determine the characteristics of CVOD appropriate for the bidirectional approach treatment.

**Methods:**

A total of 49 patients who underwent endovascular interventions with all relevant data between January 2011 and December 2015 at the First Affiliated Hospital, Sun Yat-sen University, Guangzhou, China, were included in this retrospective study, and were categorized into two groups: the 19 patients in group 1 had undergone percutaneous transluminal venoplasty (PTV) via a conventional technique (unidirectional procedure from the vein distal or proximal to the obstructive lesion), and the 30 in group 2 had undergone flossing wire technique (bidirectional procedure from femoral vein and the vein distal to obstructive lesion and using a flossing wire technique). The technical success rate, the fluoroscopy time in the procedure, perioperative complications, and patency were evaluated retrospectively.

**Results:**

Compared with group 1, group 2 had a higher initial technical success rate (83.33% vs. 47.36%, *p* = 0.012) but a shorter fluoroscopy time (82.6 ± 26.1 vs. 116.1 ± 42.1, p = 0.048). Receiver operating characteristic (ROC) analysis indicated that a lesion with a length of 6.5 cm was the best predictor of technique success (*p* = 0.02) in group 1, but no cut-off value was identified for group 2. There were no significant differences in perioperative complications between these two groups. The complication rates were 31.58% (6/19) in group 1 and 6.67% (2/30) in group 2, (*p* = 0.043), respectively. No significant difference was observed between these two groups with respect to the stent patency rate.

**Conclusion:**

Compared with the conventional technique, the flossing wire technique has a higher success rate, shorter fluoroscopy time, fewer complications and similar patency rate. It is a feasible treatment for CVOD, especially for long obstructive lesions.

## Background

Central venous occlusive disease (CVOD) is a challenge frequently encountered in the treatment of hemodialysis [1]. CVOD is often caused by the placement of a central vein catheter; however, other etiologies have also been mentioned [1–4]. CVOD often develops rapidly, manifesting as increasing massive edema in the ipsilateral arm or neck, aneurysmal dilation of the fistula, and prolonged bleeding times after needle removal, which is often accompanied by the progressive failure of the fistula [1]. It is important to conduct a timely and effective treatment for CVOD with the goal not only of relieving the patient’s symptoms, but also of maintaining the function of AVF or AVG.

While the optimal management of CVOD remains under debate, surgical approaches have been used to increase the availability of access sites and relieve symptoms, and were reported to have a primary patency rate of 80–90% after one year [5]. However, surgical approaches require general anesthesia and exhibit a high rate of surgical morbidity when used in cases with end-stage renal disease. As an alternative to surgical treatments, endovascular interventions have been performed for over 10 years and have obtained appreciable success rates and benefits, although repeated interventions have still been required in certain cases [6–8].

At present, there is no generally accepted guideline for CVOD intervention. The traditional intervention technique is typically a single-direction (unidirectional) single approach (single access) technique, which may achieve a higher success rate for vein stenosis or short occluded lesions. The flossing wire technique (or the through-and-through wire technique) has occasionally been reported in the literature, but its clinical application was only limited to complex cases. The sample size in all prior published studies was limited, and no study has explored the advantages of this technique or what time is appropriate for employing this technique on patients. On the other hand, different studies reported various success rates, as various medical centers had different levels of technical control and treated patients with various severity of CVOD [1, 6, 9–14].

The current study was conducted to retrospectively evaluate the clinical feasibility of using a flossing wire for treating complete CVOD based on 5 years of data stored in our center, and was intended to identify major factors which had limited the success rate of endovascular treatment and discuss the potential value of flossing technique in dialysis patients with special conditions.

## Methods

### Cases

From January 2011 to December 2015, 89 CVOD patients with hemodialysis fistula had been referred to our medical center for venographic analysis and endovascular treatment due to arm or neck swelling or elevated venous pressure of fistula outflow. Forty-nine cases with full relevant information for this study were enrolled (28 males and 21 females; mean age 56.5 years, age range 29–81 years). The protocol of this study was approved by the ethics committee of the First Affiliated Hospital of Sun Yat-Sen University. The study was conducted in accordance with the Declaration of Helsinki and other international guidelines. Informed consent from individual participant was waived by the committee due to the retrospective nature of this study. All medical records were reviewed retrospectively, and the details of each intervention were obtained by reviewing radiologic reports and venograms. All enrolled cases, presented with location, type, and degree of central venous occlusion, were confirmed by computed tomography, ultrasound, and venography. The standards and definitions used in this study were described in detail below with the reference of the National Kidney Foundation-Dialysis Outcomes and Quality Initiative (NKF-DOQI). The patients’ demographics, risk factors and indications for intervention are displayed in Table 1.

**Table 1:** Comparison of demographics, risk factors and indications for intervention in patients between single and dual access groups

Demographics	No, (%) or Mean ± SD (range)		
	Group 1	Group 2	*p* Value
Number of patients (n)	19	30	
Age, years	57.03 ± 10.9	55.68 ± 11.69	0.684
Sex			
Male, n	12	16	0.773
Female, n	6	14	
Diabetics			
Diabetic, n (%)	8(42%)	16(53%)	0.561
Non-diabetic, n (%)	11(58%)	14(47%)	
Duration of symptom (M)	4.4 ± 1.98	5.1 ± 1.81	0.213
Duration of dialysis (M)	55.6 ± 11.69	57.0 ± 10.92	0.689
Age of fistula (M)	14.6 ± 7.57	19.7 ± 10.26	0.056
Type of fistula			
AVF, n (%)	6 (32%)	16 (53%)	0.155
AVG, n (%)	13 (68%)	14 (47%)	
History of CVC	17 (89%)	29 (97%)	0.551
Indication for intervention			
Swelling, n (%)	17 (89%)	30 (100%)	0.145
High venous pressure, n (%)	14 (73%)	25 (83%)	0.480
Clotted access, n (%)	3 (15%)	3 (10%)	0.665
Non-specified, n (%)	1 (5%)	0 (0%)	0.388

### Standards and definitions

#### Definition of patency rate

Patency rates were defined according to the Committee on Reporting Standards for Arterio-Venous Accesses of the Society for Vascular Surgery and the American Association for Vascular Surgery. Patency rate was defined as a patent interval without recurrent stenosis or the need for further intervention after device implantation.

#### Definition of technical success

Technical success was defined as a complete whole primary procedure of PTA and deployment of stent, with no evidence of early failure. Early failure was defined as an inability to cross the lesion at the time of the primary procedure, by the presence of an occlusion, or ≥50% restenosis within the first 30 days after the initial procedure.

#### Definition of lesion length

Lesion length was defined as the distance from the definite proximal to the distal shoulder of the lesion in the projection that best elongated the occlusion. Regardless of which method was used,simultaneous proximal and distal angiogram of all lesions were performed as the first step in the endovascular treatment, and only a portion of the complete occlusion was quantified in our study (see below).

### Techniques

#### Diagnostic venography

All procedures in this study were performed by the same medical team. Diagnostic digital subtraction venography from the outflow vein of the fistulas to the right atrium was achieved by ultrasound-guided percutaneous puncture of fistulas using an 18G trocar and power contrast injection in all patients. No peripheral venous outflow occlusions were identified in these patients outside of the central venous occlusions. After primary identification of the distal end of the central vein occlusion lesion, a second percutaneous puncture access in the femoral vein was set up with a 4 F micropuncture kit (Terumo), followed by the insertion of a 4 F multipurpose angled (MPA) catheter (Cordis) to the proximal end of the occlusion lesion. Further venography by synchronous power contrast injection from both ends of the lesion with high magnification in multiple projections was performed again to identify the length and position of the occluded segment, and to determine the diameter of the normal vein close to the lesion.

#### Conventional technique procedure

**Fig. 1:**
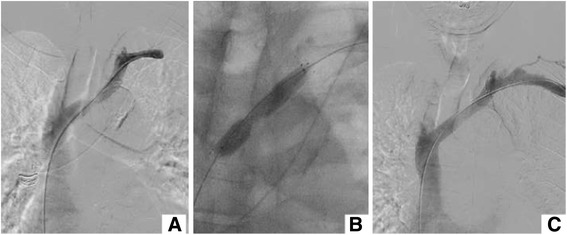
Single venous access approach through femoral vein. This case had an occluded short segment on left innominate vein. **a**. Angiography showed a 3 cm occluded segment, and catheter was advanced directly through the occluded point to the distal end via femoral vein. **b**. Stent was released on the occluded segment and balloon was dilated. **c**. After successful stent placement, angiography showed complete restoration of blood flow

**Fig. 2:**
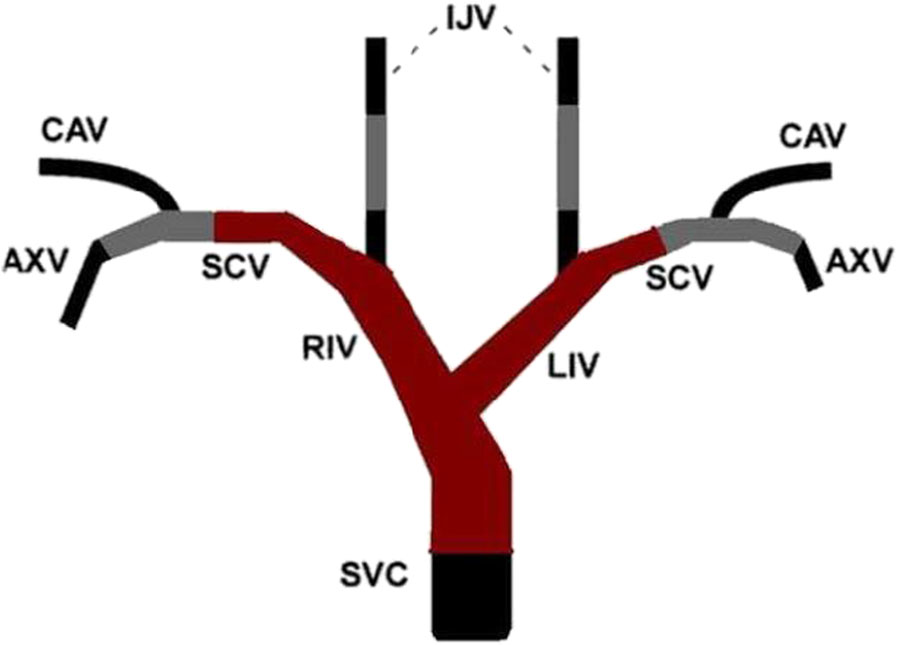
Site map of central venous occlusion disease (CVOD). Positions in red represent places of high incidence of CVOD; positions in gray are the ones frequently selected as venous access sites (“the third way”). RIV, right innominate vein; LIV, left innominate vein; LSCV, left subclavian vein; RSCV, right subclavian vein; SVC, superior vena cava; EJV, external jugular vein; IJV, internal jugular vein; CAV, cephalicarch vessel; AXV, axillary vein

**Fig. 3:**
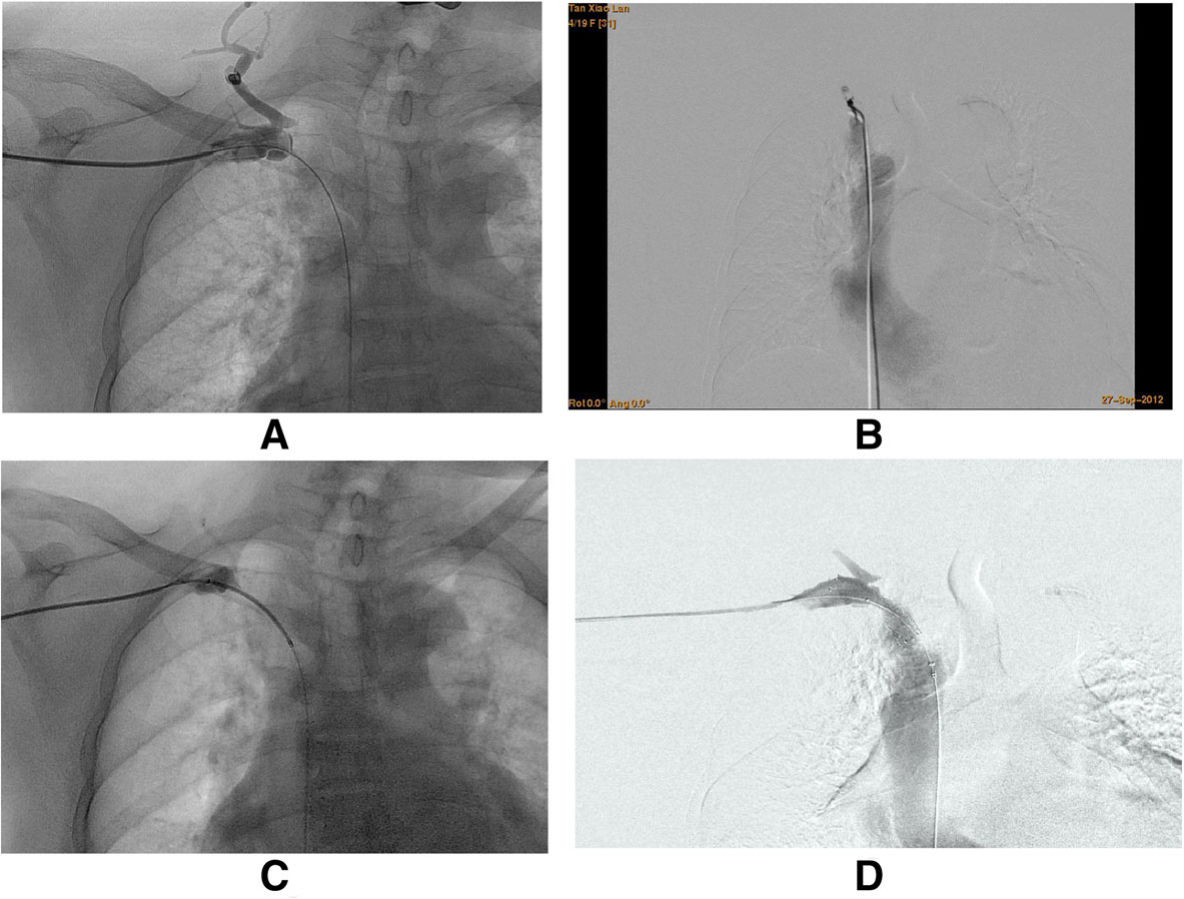
Recanalization of the distal occlusion. This case had an occluded segment on the right innominate vein. **a** Direct puncture followed by the insertion of a 6Fr sheath was performed on the distal occluded segment, and angiography showed the occlusion of the distal end. **b** Femoral vein angiography showed the occlusion of the proximal end. **c** Stent was successfully inserted after guide wire was advanced through distal end of occluded segment. **d** Angiography showed complete restoration of blood flow thorough previously occluded segment

In patients of group 1, main endovascular intervention was performed via one access regardless of the number and site of the punctures that had been performed during the full procedure. In this group, three different accesses were adopted to introduce the recanalization devices: 1) installing a femoral vein access as the main recanalization approach (Fig. 1); 2) fistula access: a 4 F Terumo sheath was inserted through the first puncture access of fistula described above; 3) generating a new intervention access with a 4 F Terumo puncture kit in the upper arm or the clavicular region, guided by ultrasound or venography in a position about 7–10 cm distal distance from the occlusion lesion, and we always called this access as “the third way” (Fig. 2). The criteria for the selection of the optimal access approach were based on the lesion characteristics. We always preferred choosing the femoral vein access or the fistula access first. However, if the lesion could not be crossed successfully, “the third way” must be considered (Fig. 3). After intervention access was installed, two technical phases, crossing the occlusion and recanalization, were performed as followed. In the first phase, a 4Fr 65-cm-long MPA catheter (Cordis) with a 0.035-in., 3-mm J tip, 135-cm-long hydrophilic Glide wire (Terumo) was inserted to cross the occlusion lesion, followed by the change of the wire from a hydrophilic Glide wire (Terumo) wire to a 260-cm length, 0.035-in. Super Stiff Amplatz (Meditech/Boston Scientific) or Super Stiff hydrophilic wire (Terumo). Out of all 19 patients, the procedure was successfully performed in 15, including 7 patients with fistula access, 3 patients with outflow venous access, and 5 patients with femoral vein access. Four patients had a failed procedure and eventually required a surgical solution. The next phase-recanalization was continued after 1^ST^ phase have been completed. The venous access sheath(s) in the successful 15 cases was then exchanged for an 8 F, 5-cm-long Terumo sheath, followed by a stepwise pre-dilation procedure. Briefly, a number of high-pressure balloons (with diameters ranging from 6 mm to 12 mm, step by step) was used to dilate the central vein occlusion. In 7 patients, the dilate balloon was pushed smoothly through the lesion. In other 8 patients, the balloon did not cross the central vein occlusion with the above mentioned approach. In these 8 cases, the sheath of intervention access site was exchanged for a 5 F, 30-cm-long Abrahams sheath with an angled tip (Cook), with the tip of this sheath being placed as close to the occlusion as possible. In 2 of these 8 patients, the balloon was finally pushed through the lesion site successfully. Among the remaining 6 patients, 3 required the use of the flossing wire technique, but these were not included in group 2 to avoid data duplication, and the remaining 3 had surgical interventions. Prior to stent deployment, the length of the stent was determined based on the length of the lesion, which was obtained from extensive power-injector central venous system venography. All patients received one or two stents when there was a central venous occlusion with suboptimal PTA, despite complete effacement of the lesion after PTA.

#### Flossing wire technique procedure

**Fig. 4:**
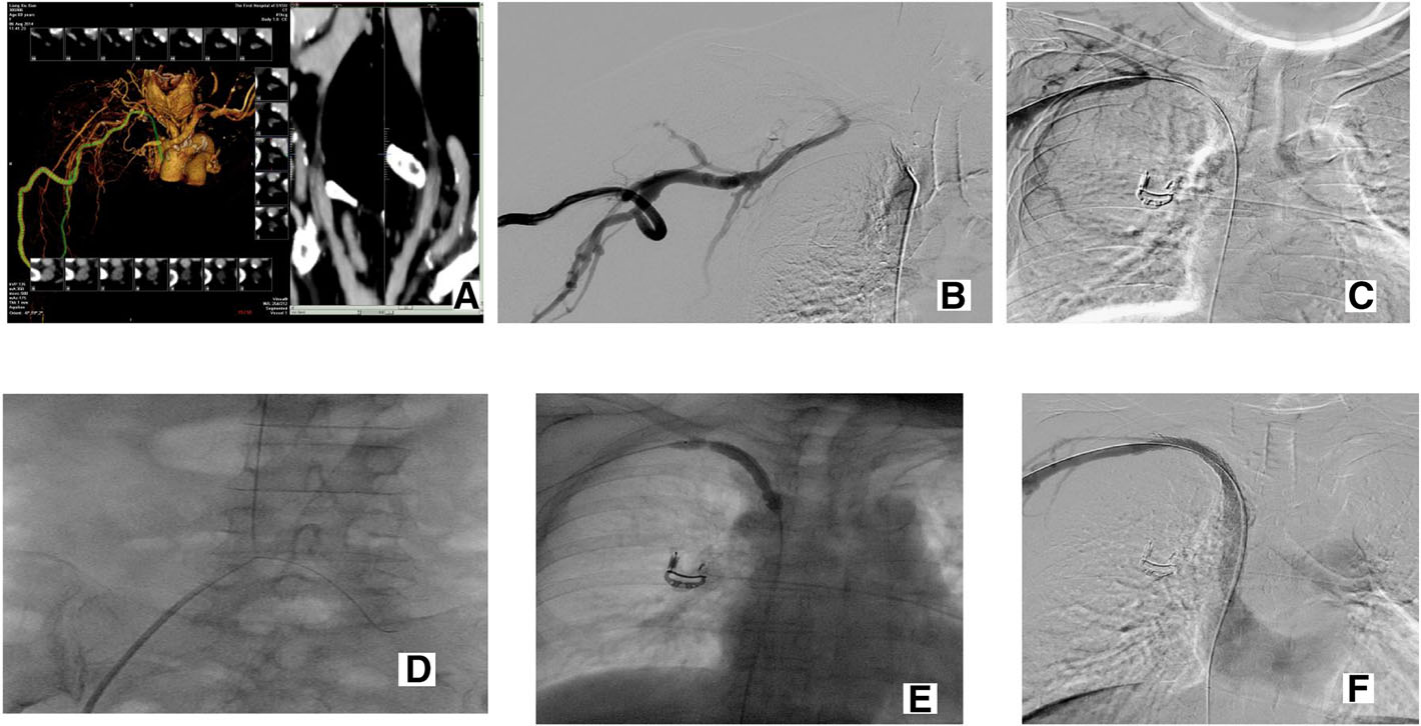
Dual venous access approach for a short occluded segment. The case had an occluded right innominate vein. **a** Preoperative evaluation of the length and extent of the occlusion by CT. **b** Synchronized angiography of the bilateral occluded ends further confirmed the length of occluded segment. **c** Guide wire was advanced via distal end of occluded segment. **d** Loop was pulled out from femoral vein sheath. **e** Balloon was dilated on the occluded segment. **f** Angiography showed successful recanalization after stent release

**Fig. 5:**
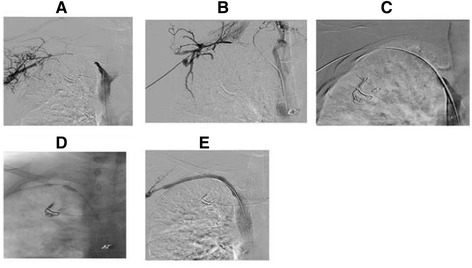
Dual venous access approach for a long occluded segment. This case had occluded segment from right subclavian vein to right innominate vein. **a** Synchronized angiography of bilateral occluded ends showed occlusion length and site. **b** Blunt recanalization from both ends of the occluded segment was performed. **c** Guide wire was advanced via distal end of occluded segment and crossed occluded segment. **d**. Balloon pre-dilation was performed segment by segment. **e** Angiography showed successful recanalization after placement of two stents

In this group, the femoral vein was defined as the main access to introduce the balloon and stents, but we also set up another access to introduce the working guidewire and perform the crossing step mentioned in the following part; this access was selected between the two remaining access points mentioned in conventional technique. The dual-access bidirectional flossing wire technique still needed the two procedure phases used in the conventional technique - crossing the occlusion and recanalization, but the difference lay in that the occlusion was crossed via distal access, and that the stent was placed via the femoral access. The first step was basically similar to the one mentioned above. After the catheter and guide wire crossed the lesion from distal to proximal, a loop snare created by a 0.18-in. guide wire (Cook) and an 8–10 Fr sheath (Terumo), and the loop was placed in the junction between inferior vena cava and femoral vein. Thereafter, with the assistance of an MPA catheter, a hydrophilic Glide wire was inserted into the femoral vein to meet the loop, and a sufficiently long segment was pulled out of the femoral sheath in preparation for the following interventions, and a short segment of the other end of the wire was retained and stretched out of the access to the fistula. Thus, a through-and-through circuit was set up. Out of the 30 patients of group 2, this procedure was unsuccessful in 7. Therefore, a new intervention access with a 4 F or 5 F Terumo puncture kit in an upper arm or clavicular region in a position about 7 cm distal to the occlusion lesion was installed, as mentioned in single technique section, and a trocar needle kit for PBD with angled metallic cannula (Cook) was selected for sharp recanalization. The hard end of a 0.018 guide wire was used as a puncture needle. Once the wire crossed the lesion, a snare was exchanged for the balloon and used to capture the wire. By stretching the wire, the metallic cannula was exchanged with a 4 F MPA catheter (Cordis), and pushed through the lesion, followed by an exchange for a 0.035-in. Super Stiff 300-cm-long hydrophilic Glide wire (Terumo), which was captured by the snare and pulled out of the sheath of femoral. This procedure was completed successfully in 2 of the 7 patients. The remaining 5 patients turned to surgical solutions. After the through-and-through circuit had been set up, a 10–12-mm-diameter, 80-mm-length balloon was introduced through the femoral sheath into the occlusion directly, and was dilated in the lesion for 2–3 min. A small balloon with a fine shape was not required for pre-dilation in this procedure (Figs. 4 and 5). This step was completed successfully in all 25 patients whose through-and-through circuit had been installed, and stent deployment followed.

#### Stent selection

Each CVOD patient received at least one self-expandable stent following the venoplasty procedure. The length of each stent was 10–20 mm longer than the lesion, and its diameter was 10%–20% larger than that of the adjacent non-affected vein. The deployed stents had a mean length of 73.9 mm (range 60–80 mm), and a mean diameter of 10.6 mm (range 8–14 mm). The placed stents included four Wallstents (Boston Scientific; Boston, MA, USA), eighteen Protégé stents (ev3 Inc.; Plymouth, MA, USA), nine Sinus stents (Optimed; Germany), seven Zilver stents (Cook Medical; Bloomington, IN, USA), and nine Viabahn (W.L. Gore & Associates INC; Arizona, U.S.A). Some patients were treated by two or three stents. No significant difference was observed with regard to the different stents used in these two groups (*p* = 0.35).

### Statistical analysis

All statistical analyses were performed using IBM SPSS Statistics for Windows, Version 19.0 (IBM Corp.; Armonk, NY) and the results were reported as the mean ± standard deviation (SD). Fisher’s exact test was used to compare patient demography and the locations of the lesions. The ROC curve was used to define the cut-off value of the lesion length affecting the technique success. Bivariate analysis was performed by the Chi-square test, and multiple analysis was performed by Cox regression. A *p* value < 0.05 was considered statistically significant. Hemodialysis access and stent patency rates were calculated using Kaplan–Meier analysis.

## Results

### Patient population

Nineteen patients (group 1) with CVOD underwent single vein access PTV, while 30 (group 2) underwent PTV performed using the flossing wire technique. The participants of the two groups showed no significant differences in terms of demographics or the characteristics of the length of the occlusive lesions (Tables 1 and 2).

**Table 2:** Comparison of characteristics of occluded lesions of patients between single and dual access groups

		Group 1	Group 2	*p* value
Lesion length		6.89 ± 3.69	7.87 ± 3.20	0.259
Lesion location	RIV	1	2	0.987
	RSCV	0	2	
	SVC	1	3	
	IJV+ SVC	1	0	
	RIV + SVC	3	4	
	RSCV + RIV	2	3	
	RSCV + RIV + SVC	2	3	
	LIV	2	3	
	LSCV	1	2	
	LIV + SVC	2	3	
	LSCV + LIV	3	3	
	LSCV + LIV + SVC	1	2	

*RIV* right innominate vein; LIV left innominate vein; *LSCV* left subclavian vein; *RSCV* right subclavian vein; *SVC* superior vena cava; *EJV* external jugular vein; *IJV* internal jugular vein; CAV

### Success rate

An initial single femoral venous access PTV procedure was successfully performed in 9/19 cases (47.36%), and a through-and-through PTV technique was performed in 25/30 patients (83.33%). The overall PTV success rate in the flossing wire group was significantly higher than that in the unidirectional technique group (p = 0.012), although the success rate of the two groups showed no significant differences in special locations of lesions, such as in RIV + SVC, RSCV + RIV, and RSCV + RIV + SVC, probably due to the limited sample size for each category (Table 3).

**Table 3:** Dual access group had a higher success rate than the unidirectional technique group

Locations of lesion	Group 1	Group 2	*p* value
RIV	(1/1) 100.00%	(2/2) 100.00%	-
RSCV	(0/0) 0.00%	(2/2) 100.00%	-
SVC	(1/1) 100.00%	(3/3) 100.00%	-
IJV+ SVC	(1/1) 100.00%	(0/1) 0.00%	-
RIV + SVC	(0/2) 0.00%	(3/4) 75.00%	0.350
RSCV + RIV	(0/2) 0.00%	(2/3) 66.67%	0.361
RSCV + RIV + SVC	(1/2) 50.00%	(2/3) 66.67%	0.709
LIV	(1/2) 50.00%	(2/2) 100.00%	0.171
LSCV	(1/1) 100.00%	(2/2) 100.00%	-
LIV + SVC	(1/2) 50.00%	(3/3) 100.00%	0.171
LSCV + LIV	(2/3) 66.67%	(2/3) 66.67%	-
LSCV + LIV + SVC	(0/1) 0.00%	(1/2) 50.00%	-
Total	(9/19) 47.36%	(25/30) 83.33%	0.012

**Fig. 6:**
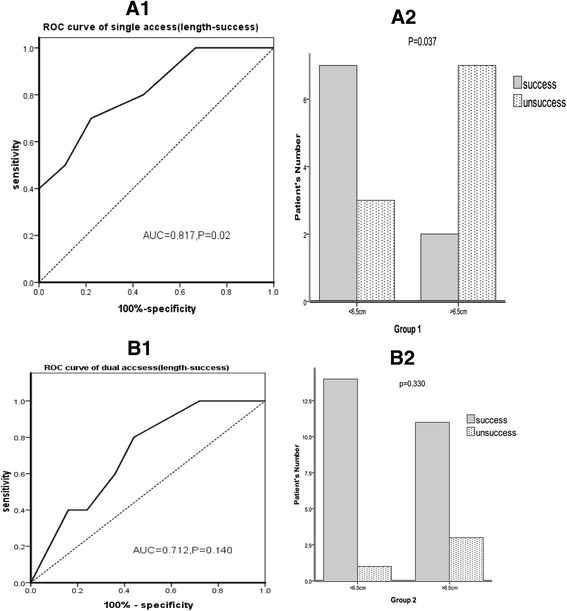
Receiver-operating characteristic (ROC) curves for determination of cutoff value of lesion length and comparison of the success rate between signal access and dual access groups. A1. In group 1 (unidirectional technique group), the cut-off value of lesion length was predicted to be 6.5 cm, with 70% sensitivity and 78% specificity, respectively. A2. Chi-square test showed a significant difference of success rate between the length of lesion which was shorter and longer than cutoff value of 6.5 cm in group 1. B1. In group 2 (dual access group), lesion length had no significant influence on technique success rate. B2. Technical success rate was not significantly linked to the length of lesion in group 2. AUC, area under ROC curve

Our ROC analysis identified lesion length as a risk factor for the technical success in the single-access venoplasty group. The cut-off value for the lesion length was 6.5 cm, as calculated using the Youden Index (Fig. 6a 1, 2). However, there was no significant correlation of the lesion length with the technical success rate of the dual access venoplasty (Fig. 6b 1, 2).

### Fluoroscopy time

Among the patients who were treated successfully with either single or dual access, the fluoroscopy time of the whole procedure was significantly different between those in group 1 and 2 (116.1 ± 42.1 vs. 82.6 ± 26.1 min, respectively, *p* = 0.048) (Table 5). We further divided the whole procedure into two steps, the wire crossing the lesion and PTAS. It showed that the difference of wire cross was not significant between these two groups (55.6 ± 15.9 vs. 59.8 ± 21.3 min, respectively, *p* = 0.540), but the difference in the time spent in performing PTA and stent deployment was significant (60.6 ± 39.95 vs. 22.8 ± 8.5 min, respectively, *p* = 0.022) (Table 4).

**Table 4:** Dual access group had a shorter fluoroscopy time than unidirectional technique group during intervention procedure

	Group	N	Mean	SD	*p* value
Tcrossing	1	9	55.5556	15.8989	0.540
	2	25	59.8000	21.2858	
Trevascularization	1	9	60.5556	39.9566	0.022
	2	25	22.8000	8.54888	
Twhole	1	9	116.1111	42.1143	
	2	25	82.6000	26.0656	0.048

Tcrossing: amount of time spent in crossing the wire; Trevascularization: amount of time spent in revascularization; Twhole: total amount of time spent in completing the entire procedure

### Post operation complications

Complications related to the operations were followed and recorded within 1 month after the operation in all patients who had undergone endovascular treatment (regardless success or not). The incidence rate was 31.58% (6/19) in group 1 and 6.67% (2/30) in group 2 (*p* = 0.043), respectively, suggesting that the dual access approach significantly reduced complications compared with the signal access approach. The complications in group 1 were hematoma at puncture position (2 patients), thrombus and dysfunction of access (2 patients), and acute heart failure (1 patient). The complications in group 2 were acute pericardial effusion (1 patient) and acute heart failure (1 patient).

### Patency rate

The mean follow-up time was 13.85 months. There were no significant differences between these two groups with respect to stent patency rate (Table 5).

**Table 5:** Dual access group had higher patency rates of hemodialysis access than the unidirectional technique group

Patency	Time	Primary patency rates (%)			Secondary patency rates (%)		
		Group 1	Group2	P Value	Group 1	Group2	*P* Value
Stent	6 m	76.92%	90.48%	0.348	100%	95.24%	1.00
	12 m	50.00%	53.85%	1.000	75.00%	84.61%	0.618

## Discussion

CVOD is the most common major problem encountered in vascular access [6–8]. Without a timely and effective management, CVOD usually results in a decrease in longevity and quality of life in patients.

Currently, endovascular therapy is the preferred method for treating CVOD, because surgical options are associated with significant morbidity and are used as alternative treatments only for patients refractory to percutaneous endovascular treatment. However, reported technical success rates, complications, and long-term outcomes of endovascular therapy varied greatly [1, 6, 9–14], which can be attributed to differences in recanalizing technique, study methodologies, patient demography, and certain characteristics of obstructive lesions including size and elasticity, as well as the degree of obstruction [1–4, 11, 15]. According to reports, technical success rates have been shown to have a range from 70% to 100% [1, 16]. However, we noted that clinical practice guidelines, such as the Kidney Disease Outcomes Quality Initiative (KDOQI) and the European Best Practice Guidelines (EBPG), were developed in 2002–2006 in developed countries [17, 18]. Therefore, in these countries, CVOD can be diagnosed and treated timely. Although not all CVS need to be addressed, because some studies suggest that there is no close correlation between CVS-induced abnormal hemodynamic changes and clinical symptoms and only 33% of CVS patients manifest clinically, [19]. There is no controversy about the necessity of complete occlusion of central vein (CVOD) with symptoms.

In China, there are currently no general guidelines for the monitoring of hemodialysis patients. Therefore, COVD is rarely diagnosed until patients are unable to receive hemodialysis via vascular access or have severe upper limb edema that significantly affects daily life, which led to a situation in which most CVS patients had progressed to completely occlusion. It makes us available to screen the enough patients with complete vascular occlusion rigidly [20, 21]. Different from most of previous studies in which CVOD was defined as >50% stenosis and/or actually included some CVS patients, our definition of CVOD was strictly defined as complete vascular occlusion, where the original blood flow of outflow vein is completely blocked before treatment [22].

Endovascular treatment of CVOD or CVS has been previously reported, but the factors limiting technical success received little attention [6–14]. Our study analyzed the technical aspects in the cases of technical failure and found two major aspects accounting for technical success. These two aspects were “crossing” and “revascularization.” Crossing is to pass the guidewire through the occlusion, and revascularization is to position the balloon and stent and angioplasty on the occluded site. “Crossing” has been recognized as key for technical success as previous reports. The difficulty of crossing the wire is related to certain characteristics of obstructive lesions including size and elasticity. There are three methods to tackle this issue: blunt crossing, sharp crossing, and balloon puncture crossing [10–13, 23, 24]. As long as the guidewire crosses the occlusion, the channel for recanalization is established, and the second step will follow. There are many ways for the balloon and stent to advance through the occlusion, including the replacement of super stiff guidewire, use of a long sheath, and flossing wire techniques. The occlusion length, extent and location are main factors influencing the resistance to passing the balloon through the occlusion. Our study showed that this second step of the operation was also the key to the success of the intervention. For instance, in all 34 successful cases in two groups, the entire average fluoroscopy time was 101 min, of which the first step took an average of 57 min, and the second step took an average of 41 min, accounting for 56% and 40% of the entire operation time, respectively. Second, in 9 of 15 cases of treatment failure, the first step was not completed, and 6 cases did not pass the second step, accounting for 60% and 40% of the total failure number, respectively. Thus, the second step contributed significantly to the overall operation time and the failure rate. In addition, based on the literature and our study, during the wire-crossing process, the actual use of a sharp crossing or balloon puncture technique was not frequent in successful cases. In 40 cases with successful crossing the wire (i.e., the first step), 2 cases had a sharp crossing technique. However, in 9 cases which failed, 1 used a sharp crossing. In 14 successful cases reported by Kundu et al., only 1 case used a sharp needle [25]. Therefore, we believe that using a sharp crossing technique had no significant impact on the overall success rate, because it was not frequently needed in the cases reported. In contrast, in the second step, a single approach super-stiff guidewire replacement and long sheath support assistive technologies were used for some of our patients, and a flossing wire technique was used for some others. Only the latter obtained a 100% success rate, suggesting that the flossing wire technique is the most effective measure to solve difficulties encountered during the second step. In addition, although some patients showed failure in the first technique and were successfully switched to the second, these patients were not included in this study to avoid interference by data duplication.

The flossing wire technique was reported to treat central venous occlusion in 1996, and has been consistently used since then [26–30]. In previous reports, the flossing wire technique was not listed as an individual technique but was used in handling complicated cases, as reported by Kundu and Haage [25, 31]. In our study, this technique was used for 30 patients, and we obtained and analyzed more data related to the use of this technique. Our findings suggest that flossing wire technology significantly reduced fluoroscopy time, and obtained a high success rate and low complication rates.

Our findings suggest that the use of the flossing wire technique can increase the surgical success rate through a better approach to handle the issue of balloon crossing the occlusion, which may be attributable to the following aspects: first, it solves the issue occurring when a wire guiding balloon through the occlusion meets resistance, rolls back, and is twisted. The length of the occluded segment is considered as the primary factor affecting the technical success rate; it affects both guidewire crossing the occlusion and balloon catheter crossing stenosis [32]. However, for the time being, no unified length-measurement method has been adopted. We used a synchronous bilateral fluorescein imaging technique to determine the length of the occluded segment, which fully presented the original information of the occluded segment prior to intervention. Our studies have shown that 6.5 cm is a critical length of occluded segment for a unidirectional technique approach. If the occlusion is longer than 6.5 cm, treatment success rate will be significantly compromised, because the long occluded segment increases the resistance to a certain level, such that the forced advance of the guidewire and balloon catheter will be rolled back and twisted. In our unidirectional technique group, 8 cases of failure had a length of occlusion longer than 6.5 cm, of which 4 cases failed during second step. Some studies used long sheaths to increase the chance of success [10, 25] as we did in our study. However, we did not succeed with this approach in patients with a large lumen diameter of blood vessel localized before and after the occluded segment. In addition, when the occlusion occurs at the turning point, direction change is the factor that resists the advance of balloon catheter, because a long sheath only increases propulsion but does not provide steering force. Flossing guidewire technology has the advantage of offering sufficient propulsion and steering force, which is the major reason why endovascular treatment has been widely used in recent years. For example, the flossing guidewire technique is efficient and useful to guide the stent through the places of extreme bending, such as the aortic arch in aortic dissection, usually referred to as the "Gothic arch" [3, 33–35]. The “Gothic arch” also exists in the central venous structures. To pass a balloon through the "Gothic arch" occlusion without the guiding force provided by the stretching guidewire is difficult, and it will also be difficult for those stents with poor compliance, the latter of which may steer away from the original direction and may hence pierce the wall of the blood vessel. Our preliminary data showed that for the lesions in certain places, such as the right innominate vein, the cross-left innominate vein, or the superior vena cava, using through-and-through technology tended to achieve a higher success rate.

In addition, the data from the follow-up with the patients with successful operations in these two groups showed no significant differences in the primary patency rates (%) and secondary patency rates (%). Thus, from the technical point of view, the improved flossing guidewire technique did not affect the long-term therapeutic effects of the patients who had been treated successfully. However, the flossing wire technique has advantages with regards to short-term complications. The unidirectional technique approach has two options, from either the proximal or the distal end of the occluded segment. For the occlusion of the long segment, performing recanalization from the distal end is preferred because the distance is shorter and the operation is more controllable [10, 14]. This approach usually takes a shunt or draining vein as a channel for the import of various interventional devices, and requires a catheter sheath with a size of 8Fr or even larger, which has a high risk of generating hematoma [36]. Although the pressure of the shunt and outflow vein differs in AVF and AVG. The effective ΔP in the fistula generally is only 8 to10 mm Hg, frequently 25%, and seldom more than half those noted in grafts [17]. Given that many patients with kidney diseases have hypertension, hemostasis should be implemented timely and efficiently and should be given sufficient amount of time after the sheath pullout. Under the impact of extreme edema of the lesion, hemostasis performed actually will not be the same as that achieved by artery pressing. Vascular closure devices may reduce the incidence of hematoma, but may also theoretically increase the risk of draining vein stenosis [9]. In our flossing wire approach, we introduced catheter and wire through the distal access with a 4Fr vascular sheath, which significantly reduced the damage to the vascular access. We usually chose the femoral vein as another access with an 8fr or even larger vascular sheath to the balloon and stent. Femoral vein access is generally recognized as a safe passage, and the use of a large sheath has no impact on dialysis. Therefore, we did not see complications such as hematoma and dialysis access occlusion, as observed in the unidirectional technique procedure.

In the present study, we also found that the flossing wire technique had shorter fluoroscopy time than the unidirectional technique approach during the full treatment period, although it was required to generate two vascular access sites, because the guidewire stretching saved time during the second step. Using the flossing guidewire technique, the stent was managed to cross the occlusion and deployed directly. By contrast, using the unidirectional technique approach, in order to ensure that the stent crosses the occlusion and is positioned smoothly, a small balloon is usually needed for pre-dilation. Therefore, in the second part of the procedure, the flossing guidewire technique is actually succinct.

## Conclusions

There were no significant differences in the patency rate between these two approaches in our study. Previous studies showed that the nature of the lesion and the stent type (bare stent or stent graft) will affect the patency rate, but did not investigate the impact of techniques used on the patency rate. In the present study, we did not see the difference in the number of specific stenting types used in the patients of these two groups, and the comparable patency rate between these two groups suggested that the technique was not a major determinant for the patency. Therefore, we concluded that flossing wire technology is a simpler and safer treatment for CVOD, especially for the long segment occlusion. However, our study was a retrospective study with a limited sample size. Given that the success rate and patency rate are affected by multiple factors including lesion characteristics, stenting type, and patient’s compliance, our findings need to be further corroborated in the future studies with a large sample size and more data through multivariate analysis [2, 37].

## Abbreviations

AVF: Arteriovenous fistula
AVG: Arteriovenous graft
CVOD: Central venous occlusive disease
CVS: Central venous stenosis
EJV: External jugular vein
IJV: Internal jugular vein
LIV: Left innominate vein
LSCV: Left subclavian vein
MPA: Multipurpose angled catheter
NKF-DOQI: National Kidney Foundation-Dialysis Outcomes and Quality Initiative
PTA: Angioplasty
PTAS: Angioplasty with stent
RIV: Right innominate vein
ROC: Curve, receiver operating characteristic curve
RSCV: Right subclavian vein
SVC: Superior vena cava
